# IgG Suppresses Antibody Responses in Mice Lacking C1q, C3, Complement Receptors 1 and 2, or IgG Fc-Receptors

**DOI:** 10.1371/journal.pone.0143841

**Published:** 2015-11-30

**Authors:** Joakim J. E. Bergström, Birgitta Heyman

**Affiliations:** Department of Medical Biochemistry and Microbiology, Uppsala University, Uppsala, Sweden; University Medical Center of the Johannes Gutenberg University of Mainz, GERMANY

## Abstract

Antigen-specific IgG antibodies, passively administered to mice or humans together with large particulate antigens like erythrocytes, can completely suppress the antibody response against the antigen. This is used clinically in Rhesus prophylaxis, where administration of IgG anti-RhD prevents RhD-negative women from becoming immunized against RhD-positive fetal erythrocytes aquired transplacentally. The mechanisms by which IgG suppresses antibody responses are poorly understood. We have here addressed whether complement or Fc-receptors for IgG (FcγRs) are required for IgG-mediated suppression. IgG, specific for sheep red blood cells (SRBC), was administered to mice together with SRBC and the antibody responses analyzed. IgG was able to suppress early IgM- as well as longterm IgG-responses in wildtype mice equally well as in mice lacking FcγRIIB (FcγRIIB knockout mice) or FcγRI, III, and IV (FcRγ knockout mice). Moreover, IgG was able to suppress early IgM responses equally well in mice lacking C1q (C1qA knockout mice), C3 (C3 knockout mice), or complement receptors 1 and 2 (Cr2 knockout mice) as in wildtype mice. Owing to the previously described severely impaired IgG responses in the complement deficient mice, it was difficult to assess whether passively administered IgG further decreased their IgG response. In conclusion, Fc-receptor binding or complement-activation by IgG does not seem to be required for its ability to suppress antibody responses to xenogeneic erythrocytes.

## Introduction

Antibodies, passively administered together with their specific antigen, can profoundly influence the immune response against the specific antigen via antibody feedback regulation. This phenomenon is antigen specific and can cause either >100-fold enhancement or >99% suppression of the humoral response. The outcome is dependent both on the antibody class and the type of antigen involved [1, 2]. The most wellknown antibody feedback mechanism is the capacity of specific IgG to suppress antibody responses against large particulate antigens, such as erythrocytes [3–6]. The mechanism underlying IgG-mediated immune suppression against erythrocytes remains poorly understood, and, so far, no knockout mouse strain has been found in which IgG-mediated immune suppression does not work. Nevertheless, this inhibitory function of IgG has been used successfully in the clinic to prevent hemolytic disease of the fetus and newborn since the 1960s [7, 8]. This disease is caused by transplacental hemorrhage of Rhesus (Rh) D^+^ fetal erythrocytes to a RhD^-^ mother who can produce IgG anti-RhD antibodies which cross the placenta and damage fetal erythrocytes. By administration of preformed IgG anti-RhD to RhD^-^ women, immunization can be prevented.

Several hypotheses have been proposed to explain how specific IgG can suppress antibody responses. Co-crosslinking of the B cell receptor (BCR) with the inhibitory Fc gamma receptor (FcγR) IIB negatively regulates BCR mediated activation of the B cell [9]. IgG in complex with its antigen may cause such co-crosslinking and turn off the specific B cell, thus hypothetically explaining IgG-mediated suppression. However, IgG suppresses primary IgM anti-sheep red blood cell (SRBC) responses equally well in FcγRIIB-deficient (FcγRIIB KO) as in wildtype mice [4, 5] arguing against this idea. Alternatively, specific IgG could mark the antigen for rapid elimination by phagocytosis which would reduce the amount of antigen and the possibility of interaction with antigen specific B-cells. Noteworthy is that IgG suppresses primary IgM responses in mice lacking the common Fc receptor gamma chain (FcRγ KO, lacking FcγRI, III, and IV) and in β-2-microglobulin-deficient mice (lacking the neonatal FcR, FcRn) [4]. Therefore, any type of elimination of antigen would be independent of FcγRs. Owing to difficulties differentiating between passively administered and actively produced IgG, the role of FcγRIIB or FcγRI, III, and IV in IgG-mediated suppression of longterm primary IgG-responses has not been tested.

Another hypothesis is that IgG, binding to the antigen, will mask epitopes and cause steric hindrance, thus preventing specific B cells from recognizing the antigen. This idea is compatible with the majority of experimental data, such as the lack of FcγR-dependence and the ability of F(ab')_2_ fragments and IgE to suppress [4, 10]. However, it may not be the only explanation and mathematical modeling, based on available experimental results, suggests that IgG-mediated immune suppression is best described as a synergistic model involving both epitope masking and rapid antigen elimination [11]. In Rhesus prophylaxis, elimination of RhD^+^ erythrocytes by the administered IgG anti-RhD is considered to play an important role [12]. Moreover, we have observed that less SRBC is found in the marginal zone of mice immunized with IgG together with SRBC than in controls given SRBC alone [13], suggesting that the SRBC might be partially eliminated by the presence of specific IgG. In a recent study, murine transgenic erythrocytes, expressing hen egg lyzozyme (HEL) in sequence with ovalbumin and the human Duffy transmembrane protein were used as antigen instead of xenogeneic SRBC [6]. Here, both Duffy-specific mAbs (which increased clearance but did not bind to HEL) and HEL-specific IgG mAbs (which did not increase clearance but bound to HEL) suppressed the antibody response against HEL. The authors concluded that neither epitope masking nor increased clearance were indispensible for IgG-mediated suppression.

In addition to phagocytosis, SRBC may be rendered less immunogenic by complement-mediated lysis. The role of complement in IgG-mediated suppression of antibody responses has to our knowledge only been investigated in one study. Two monoclonal IgG1 antibodies, one which could and one which could not activate the classical complement pathway, were shown to suppress primary IgM anti-SRBC responses equally well [14]. Studies of the role of complement in immunosuppression is hampered by the fact that the early complement components C1q, C2, C3, and C4, as well as complement receptors 1 and 2 (CR1/2), are required for induction of normal antibody responses (reviewed in [15]). This means that the antibody response in mice given SRBC alone is very low and therefore a possible suppression of this response by passively administered IgG would be difficult to detect.

In this study we investigate for the first time whether polyclonal IgG anti-SRBC can suppress IgM- and IgG-responses in mice lacking C1q, C3, or CR1/2 and whether IgG can suppress the longterm IgG-response in mice lacking FcγRs I, III, and IV (FcRγ KO) or FcγRIIB.

## Materials and Methods

### Ethics statement

The Uppsala Animal Research Ethics Committee specifically approved this study (Permit numbers: C146/10 and C25/13). The mice were bred and maintained in the animal facilities at the National Veterinary Institute (Uppsala, Sweden). Skilled personnel under the supervision of the veterinarian in charge routinely observed the health status of the mice.

### Mice

BALB/c mice were obtained from Bommice (Ry, Denmark). Mice deficient in FcγRIIB, C.129S4(B6)-Fcgr2b^tmlTtK^/cAnNTac N12 (FcγRIIB KO) were purchased from Taconic Biosciences, Inc. (Hudson, NY, USA), FcRγ KO founders were a gift from Dr J. V. Ravetch [16] and backcrossed to BALB/c for 10 generations. Wildtype C57BL/6 mice, C57BL/6 mice lacking complement factor C3 (C3 KO) and Ig allotype congenic mice, C.BKa-Igh^b^/lcrSMnJ (CB17), were obtained from Jackson Laboratories (Bar Harbor, ME, USA). Mice lacking CR1/2 (Cr2 KO) were a gift from Dr H. Molina [17] and backcrossed for 10 generations to BALB/c background. C57BL/6 C1qA-deficient founder mice (C1q KO), lacking the classical pathway activator C1q, were obtained from Dr M. Botto [18]. Mice were age and sex matched within each experiment. All animal experiments were approved by the Uppsala Animal Research Ethics Committee. Animals were bred and maintained in the animal facilities at the National Veterinary Institute (Uppsala, Sweden).

### Antibodies

Polyclonal IgG^a^ anti-SRBC was prepared from hyperimmune BALB/c serum and polyclonal IgG^b^ anti-SRBC from hyperimmune C57BL/6 or CB17 serum. IgG was purified on a Protein-A Sepharose column (Amersham Pharmacia Biotech, Uppsala, Sweden) [19], dialyzed against PBS, sterile filtered and stored at -20°C until use.

### Antigens

SRBC were obtained from Håtunalab AB (Håtunaholm, Sweden) and stored in sterile Alsever’s solution at 4°C. SRBC were washed three times in PBS prior to use.

### Immunizations and blood sampling

Mice were immunized with polyclonal IgG anti-SRBC and SRBC in one of their lateral tail veins in 200 μl PBS. IgG anti-SRBC was given 30 min prior to SRBC. Controls received SRBC alone or IgG anti-SRBC alone. Details regarding doses and IgG-allotypes are given in the figure legends. Blood was collected from the ventral tail artery.

### Direct plaque forming cell assay

A modified version of the Jerne hemolytic plaque forming cell assay (PFC) was used to assess single cells producing SRBC-specific IgM [20]. In brief, a mixture of 100 μl spleen cell suspensions, 25 μl guinea pig serum (diluted 1:10; as source of complement), 25 μl 10% SRBC-suspension and 300 μl 0.5% 1:1 agarose (SeaPlaque low gelling temperature, FMC Bioproducts, Rockland, ME, USA) and agarose (USB corporation, Cleveland, OH, USA) was poured onto glass slides. The slides were incubated at 37°C for 3 h. Samples were counted blindly as duplicates under a magnifying glass. All dilutions were made in HBSS.

### Enzyme linked immunosorbent assay (ELISA)

The IgG anti-SRBC enzyme linked immunosorbent assay (ELISA) has been described previously [21]. In order to detect IgG anti-SRBC of the Ig^a^ or Ig^b^ allotype, a 1:1 mixture of biotinylated anti-mouse IgG1^a^ and IgG2a^a^, or anti-mouse IgG1^b^ and IgG2a^b^, was used (BD Pharmingen, San Jose, CA, USA). Plates were developed using alkaline phosphatase conjugated to streptavidin (BD Pharmingen). Unless otherwise indicated in figure legends, absorbance at 405 nm was measured after 30 min. Data was analyzed using SoftMax software (Molecular Devices. Sunnyvale, CA, USA). The results are given as OD values and serum dilution is chosen so that the highest value does not reach plateau level.

### Statistical analysis

Statistical differences between groups were determined by the two-tailed Student’s t-test. Statistical significance levels were set as: ns, p > 0.05; *, p < 0.05; **, p < 0.01; ***, p < 0.001.

## Results

### IgG-mediated suppression of primary IgM-responses in C1q KO and C3 KO mice

**Fig 1:**
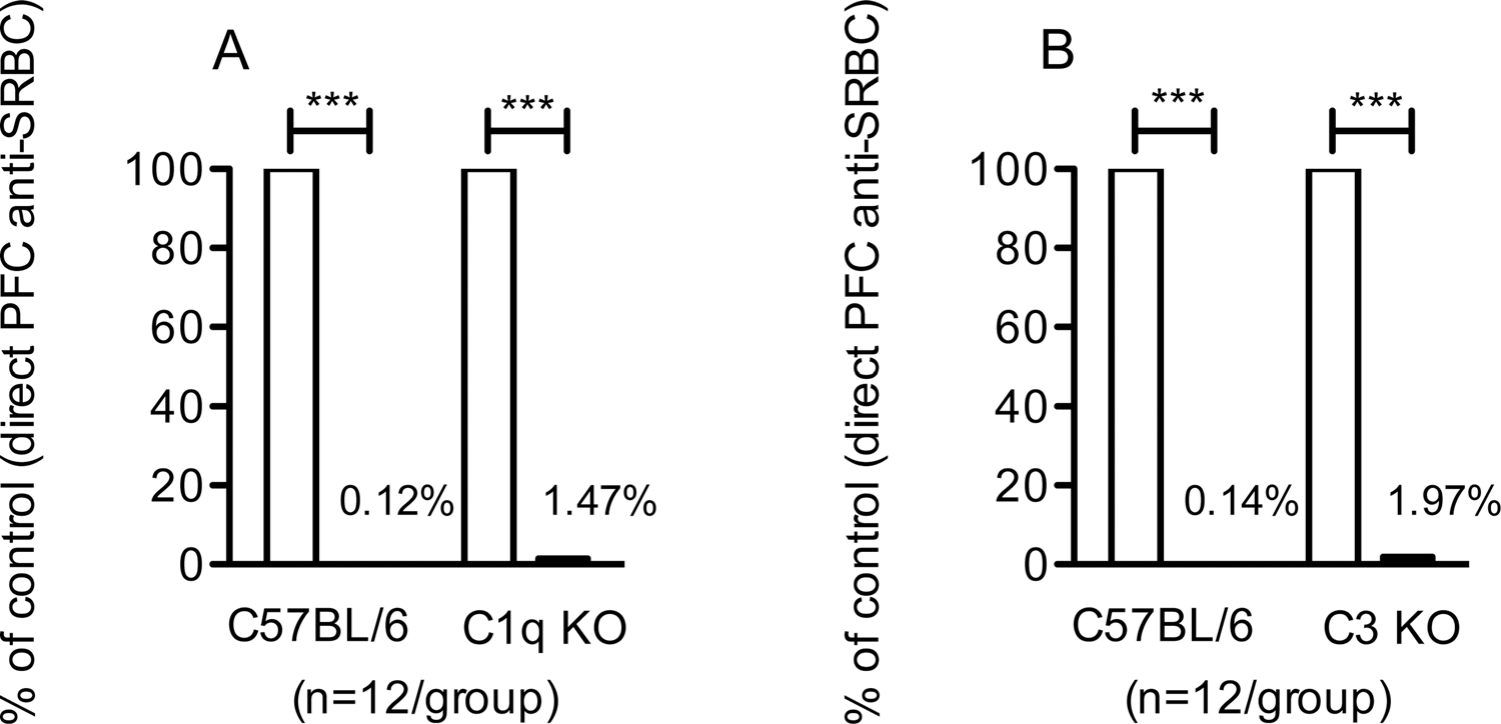
IgG-mediated suppression of primary IgM-responses in C1q KO and C3 KO mice. C1q KO, C3 KO, and C57BL/6 mice were immunized with 50 μg IgG^a^ anti-SRBC and 5x10^7^ SRBC, 5x10^7^ SRBC alone, or with 50 μg IgG^a^ alone. (A,B) Five days after immunization, the number of spleen cells producing IgM anti-SRBC was assayed. Responses are shown as percentage of the direct PFC response/spleen in mice given SRBC alone (100%, open bars); black bars show responses in mice given IgG and SRBC. Direct PFC/spleen in the respective control groups (receiving antigen alone) were in A: C57BL/6, 56,105; C1q KO, 5,433 and in B: C57BL/6, 54,954; C3 KO, 3,614. Data are pooled from three experiments; (n = 4/group in each experiment). p-values denote comparisons between mice immunized with IgG anti-SRBC together with SRBC and mice immunized with SRBC alone. ***, p < 0.001.

To study the role of complement in IgG-mediated suppression, C57BL/6 mice, C1q KO and C3 KO mice were immunized with IgG^a^ anti-SRBC and SRBC, SRBC alone or IgG^a^ alone. The passively administered IgG anti-SRBC was prepared from mice with a different IgG allotype than the recipient mice, thus facilitating analysis only of the actively produced IgG. Spleens were harvested at day 5 after immunization and single cells producing SRBC-specific IgM were assayed in a direct hemolytic plaque forming cell (PFC) assay. IgG suppressed over 98% of the IgM-responses in C57BL/6, C1q KO, and C3 KO mice (Fig 1A and 1B). As expected, IgG efficiently suppressed the IgG anti-SRBC response in wildtype C57BL/6 mice (Figure A and D in S1 Fig). C1q KO and C3 KO mice generally have very poor antibody responses [22–24] and the IgG-response to SRBC administered alone in C1q KO or C3 KO mice was extremely low when the same serum dilution and incubation times as for C57BL/6 mice were used in the ELISA (Figure B and E in S1 Fig). Testing less diluted samples and extending the substrate incubation time to 3 h in the ELISA, a detectable IgG-response appeared also in C1q KO and C3 KO mice, albeit with high background levels (Figure C and F in S1 Fig). This response was significantly suppressed day 7 and 21 in C1q KO mice (Figure C in S1 Fig). IgG appeared to suppress also in C3 mice, although the differences were not significant (Figure F in S1 Fig).

Thus, IgG efficiently suppressed the IgM anti-SRBC responses both in C1q KO and C3 KO mice. Suppression of IgG-responses were difficult to measure, owing to low antibody responses to SRBC itself, but data indicate that IgG suppressed also IgG-responses in C1q KO and C3 KO animals.

### IgG-mediated suppression of primary IgM-responses in Cr2 KO mice

**Fig 2:**
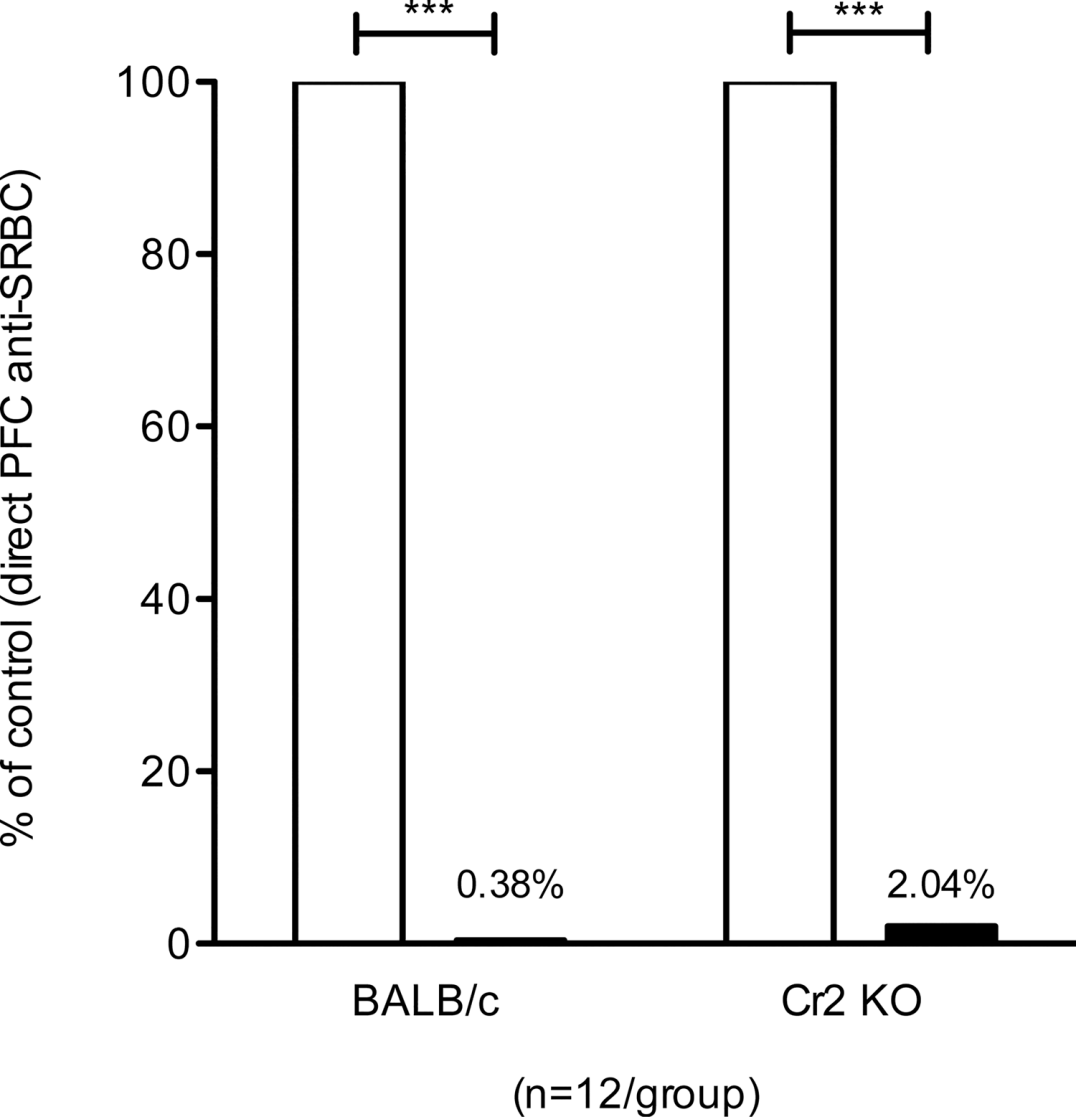
IgG-mediated suppression of primary IgM-responses in Cr2 KO mice. Cr2 KO and BALB/c mice were immunized with 50 μg IgG^b^ anti-SRBC and 5x10^7^ SRBC, SRBC alone, or IgG alone. Five days after immunization, the number of spleen cells producing IgM anti-SRBC was assayed. Responses are shown as percentage of the direct PFC response/spleen in mice given SRBC alone (100%, open bars); black bars show responses in mice given IgG together with SRBC. Direct PFC/spleen in the respective control groups (receiving antigen alone) were: BALB/c, 76,208; Cr2 KO, 11,015. Data are pooled from three experiments; (n = 4/group in each experiment). p-values denote comparisons between mice immunized with IgG anti-SRBC together with SRBC and mice immunized with SRBC alone. ***, p < 0.001.

To address whether CR1/2 are required for the suppressive function of passively administered IgG, BALB/c and Cr2 KO mice were immunized with IgG^b^ anti-SRBC and SRBC, SRBC alone, or IgG alone. IgG suppressed 98% or more of IgM-responses both in BALB/c wildtype controls and Cr2 KO mice (Fig 2) and IgG also efficiently suppressed the IgG anti-SRBC response in BALB/c controls (Figure G in S1 Fig). Cr2 KO mice have impaired antibody responses to antigens administered alone [17, 23, 25]. Therefore, as expected, Cr2 KO mice given SRBC alone produced very low levels of IgG anti-SRBC, both when assayed at a serum dilution of 1:625 with a substrate incubation during 30 min (Figure H in S1 Fig) and at a serum dilution of 1:25 with a substrate incubation during 3 h (Figure I in S1 Fig). Therefore, no conclusions regarding the ability of IgG to suppress the IgG-response in Cr2 KO mice could be drawn.

### IgG-mediated suppression of primary IgM- and IgG-responses in FcγRIIB KO mice

**Fig 3:**
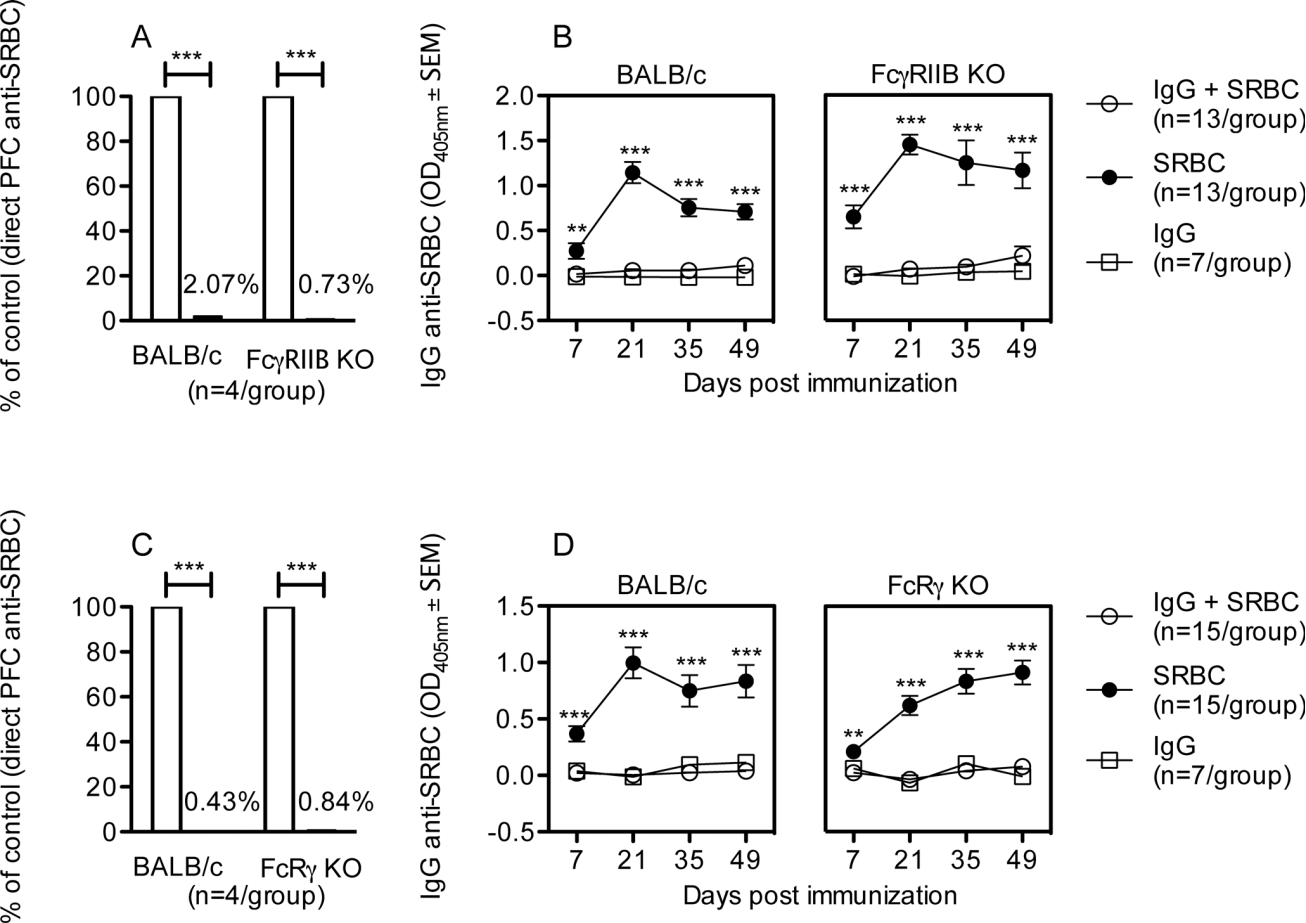
IgG-mediated suppression of primary IgM- and IgG-responses in FcγRIIB KO and FcRγ KO mice. FcγRIIB KO, FcRγ KO, and BALB/c mice were immunized with 10 μg IgG^b^ anti-SRBC and 5x10^6^ SRBC, SRBC alone, or IgG alone. (A,C) Five days after immunization, the number of spleen cells producing IgM anti-SRBC was assayed. Responses are shown as percentage of the direct PFC response/spleen in mice given SRBC alone (100%, open bars); black bars show responses in mice given IgG and SRBC. Direct PFC/spleen in the respective control groups (receiving antigen alone) were in A: BALB/c, 12,218; FcγRIIB KO, 36,391 (p<0.05) and in C: BALB/c, 57,279; FcRγ KO 40,831. (B,D) Seven to 49 days later, serum levels of IgG anti-SRBC were assayed in ELISA on sera diluted 1:625. Data are representative of one (A, C) or pooled from three (B, D) experiments with each KO strain. p-values denote comparisons between mice immunized with IgG anti-SRBC together with SRBC and mice immunized with SRBC alone. **, p < 0.01; ***, p < 0.001.

FcγRIIB KO and BALB/c mice were immunized with IgG^b^ anti-SRBC and SRBC, SRBC alone, or IgG^b^ alone. IgG efficiently suppressed the IgM response in both strains (Fig 3A). Using the allotype ELISA system described above, it was shown that IgG suppressed also longterm IgG-responses in FcγRIIB KO as well as in BALB/c wildtype controls (Fig 3B). Noteworthy is that FcγRIIB KO mice, immunized with SRBC alone, generated higher IgM anti-SRBC responses than BALB/c wildtype mice (Fig 3A legend). This finding confirms the negatively regulating effect of FcγRIIB on antibody responses previously reported [26]. In summary, efficient suppression of IgM and IgG responses by specific IgG takes place in the absence of FcγRIIB.

### IgG-mediated suppression of primary IgM- and IgG-responses in FcRγ KO mice

To investigate whether suppression of primary antibody responses to SRBC is dependent on FcγRI, III, and IV, BALB/c controls and FcRγ KO mice were immunized with IgG^b^ anti-SRBC and SRBC, SRBC alone, or IgG^b^ alone. IgM- (Fig 3C) and serum IgG anti-SRBC responses (Fig 3D) were severely suppressed by IgG anti-SRBC in BALB/c controls as well as in FcRγ KO mice. In summary, IgG efficiently suppresses both the IgM- and the IgG-response in mice lacking FcγRI, FcγRIII, and FcγRIV.

## Discussion

The molecular mechanism(s) behind IgG-mediated suppression of antibody responses to erythrocytes and other particulate antigens are important to elucidate, both because it is theoretically interesting and because IgG is used clinically in Rhesus prophylaxis. This successful therapy relies on IgG anti-RhD from large serum pools which are aquired from RhD-negative women, immunized during pregnancies with RhD- positive fetuses, or from RhD-negative males deliberately immunized with RhD-positive blood. However, owing to the success of the therapy, the number of immunized women is rapidly decreasing and ethical considerations concerning immunization of male volunteers are arising. Therefore, there is a need for therapeutic monoclonal IgG anti-RhD antibodies, but so far attempts to generate monoclonals that work well in Rhesus prophylaxis have been largely unsuccessful [27]. This may be due to the fact that it is not yet known which features of the IgG antibodies that are important for their suppressive ability.

One of the most widely discussed explanations for IgG-mediated suppression is negative regulation via FcγRIIB. This receptor undoubtedly plays a role in downregulating many effects mediated via immunoreceptor tyrosine-based activation motif (ITAM)-signalling receptors both in vitro and in vivo [26, 28, 29]. The two original studies performed on the importance of FcγRIIB for IgG-mediated suppression of antibody responses against SRBC, showed that suppression of day 5–6 IgM and IgG responses took place equally well in mice lacking this receptor as in wildtype controls [4, 5]. These results were unexpected and created some debate at the time [12, 30, 31], but were recently confirmed in a study using allogeneic RBC, expressing a fusion protein consisting of hen egg lysozyme, OVA, and Duffy b as the antigen [32]. In the present study, we extend and confirm previous data. Using an allotype ELISA, which enabled selective quantification of actively produced IgG, we show that IgG suppresses also the long term IgG response equally well in FcγRIIB KO as in wildtype mice (Fig 3B). The IgM-response in control groups, given SRBC alone, was higher in FcγRIIB KO than in wildtype mice, supporting the notion that FcγRIIB is a negatively regulating receptor. However, it appears that this receptor acts to downmodulate an ongoing immune response, rather than to completely extinguish an antibody response after passive administration of IgG. Likewise, confirming and extending previous data [4], IgG was able to suppress longterm IgG responses in mice lacking all activating FcγRs, owing to deletion of the common FcRγ chain (Fig 3D). This suggests that, should elimination of SRBC via phagocytosis play a role for IgG-mediated suppression, the process must take place without involvement of FcγRs.

The murine IgG subclasses IgG2a, IgG2b, and IgG3 all activate the classical pathway, whereas IgG1 is considered a poor complement activator [33, 34]. Rare monoclonal IgG1 antibodies are however able to activate complement, and studies of the suppressive ability of one IgG1 that could, and one that could not, activate complement showed that both were able to suppress antibody responses [14]. This study however did not address whether other IgG isotypes than IgG1 utilize complement, or whether the alternative or lectin pathways are involved. Here, we have studied IgG-mediated suppression in mice lacking CR1/2, C3, or C1q. C1q is required for classical complement activation by all IgG isotypes. Cleavage of C3 can be initiated by the classical, alternative, and lectin complement activation pathways. Thus, lack of C3 prevents generation of the membrane attack complex and therefore no lysis of e. g. erythrocytes can take place. Lack of C3 also prevents generation of C3 split products, which are the ligands of CR1/2. Although animals lacking CR1/2, C3, or C1q are known to have severely impaired antibody responses [15], the IgM responses are sometimes less affected than the IgG responses [17]. This was clearly the case in our system, and therefore we were able to demonstrate that IgG suppressed the IgM responses in these animals equally efficiently as in wildtype mice (Figs 1A, 1B and 2). As expected, the IgG-responses were very low in all complement knockout mice and it was impossible to determine any suppression after normal ELISA incubation times. After longer incubation times, data indicated that IgG suppressed IgG responses in C1q KO and C3 KO mice, whereas no conclusion could be drawn from Cr2 KO mice. Taken together, we find no evidence of reduced suppression in complement-deficient mice. This supports earlier observations [14], and argues against IgG-mediated lysis of SRBC as an explanation for IgG-mediated suppression.

The possibility that complement and FcγRs act redundantly in IgG-mediated suppression of antibody responses has not been analyzed here. Testing the question of redundancy in gene targeted mice is difficult since "multiple" KO's, lacking both complement and FcγRs, would have extremely low antibody responses. As discussed above, this precludes reliable analysis of suppression. An alternative approach would be to use deglycosylated IgG, lacking ability to bind to FcγRs as well as to activate complement[35, 36]. However, we would not expect to see a difference since F(ab')_2_ fragments, lacking both ability to bind to FcγRs and to activate complement, are efficient suppressors in vivo [4]. Moreover, IgE can suppress responses to SRBC [4], and although IgE has been reported to bind FcγRIIB and FcγIII, IgE-mediated suppression works well in mice lacking these receptors[37]. In conclusion, the mechanism behind IgG-mediated suppression remains enigmatic, but the data presented here strongly argues against involvement of either FcγRs or complement.

## Supporting Information

S1 Fig IgG-mediated suppression of primary IgG-responses in C1q KO, C3 KO and Cr2 KO mice. C1q KO, C3 KO and C57BL/6 mice were immunized with 50 μg IgG^a^ anti-SRBC and 5x10^7^ SRBC, 5x10^7^ SRBC alone, or with 50 μg IgG^a^ alone. Cr2 KO and BALB/c mice immunized with 50 μg IgG^b^ anti-SRBC and 5x10^7^ SRBC, 5x10^7^ SRBC alone, or with 50 μg IgG^b^ alone. (A-I) Seven-49 days after immunization, serum levels of IgG anti-SRBC were assayed in ELISA on sera diluted 1:625 (A,B,D,E,G,H) or 1:25 (C,F,I). Incubation times with substrate were either 30 min or 3 h. Data are representative of two (A-C, G-I) or one (D-F) experiments; (n = 3-5/group in each experiment). p-values denote comparisons between mice immunized with IgG anti-SRBC together with SRBC and mice immunized with SRBC alone. ns, p > 0.05 (not indicated); *, p < 0.05; **, p < 0.01; ***, p < 0.001.
(PDF)

## References

[1] Heyman B. Regulation of antibody responses via antibodies, complement, and Fc receptors. Annu Rev Immunol. 2000;18:709–37. 10837073 10.1146/annurev.immunol.18.1.709

[2] Heyman B. Antibodies as natural adjuvants. Current topics in microbiology and immunology. 2014;382:201–19. 10.1007/978-3-319-07911-0_9 . 25116101

[3] Henry C, Jerne N. Competition of 19S and 7S antigen receptors in the regulation of the primary immune response. J Exp Med. 1968;128:133–52. 5662011 10.1084/jem.128.1.133 PMC2138503

[4] Karlsson MCI, Wernersson S, Diaz de Ståhl T, Gustavsson S, Heyman B. Efficient IgG-mediated suppression of primary antibody responses in Fc-gamma receptor-deficient mice. Proc Natl Acad Sci USA. 1999;96:2244–9. 10051626 10.1073/pnas.96.5.2244 PMC26768

[5] Karlsson MCI, Getahun A, Heyman B. FcγRIIB in IgG-mediated suppression of antibody responses: different impact *in vivo* and *in vitro* . J Immunol. 2001;167:5558–64. 11698426 10.4049/jimmunol.167.10.5558

[6] Yu H, Stowell SR, Bernardo L, Hendrickson JE, Zimring JC, Amash A, et al Antibody-mediated immune suppression of erythrocyte alloimmunization can occur independently from red cell clearance or epitope masking in a murine model. J Immunol. 2014;193(6):2902–10. Epub 2014/08/15. doi: jimmunol.1302287 [pii] 10.4049/jimmunol.1302287 . 25122924

[7] Clarke CA, Donohoe WTA, Woodrow JC, Finn R, Krevans JR, Kulke W, et al Further experimental studies on the prevention of Rh haemolytic disease. Br Med Journal. 1963;1:979–84. 14021558 10.1136/bmj.1.5336.979 PMC2122888

[8] Urbaniak SJ, Greiss MA. RhD haemolytic disease of the fetus and the newborn. Blood Rev. 2000;14(1):44–61. Epub 2000/05/11. doi: S0268-960X(99)90123-6 [pii] 10.1054/blre.1999.0123 . 10805260

[9] Daeron M, Lesourne R. Negative signaling in Fc receptor complexes. Advances in immunology. 2006;89:39–86. Epub 2006/05/10. doi: S0065-2776(05)89002-9 [pii] 10.1016/S0065-2776(05)89002-9 . 16682272

[10] Heyman B. Antibody feedback suppression: towards a unifying concept? Immunology Letters. 1999;68:41–5. 10397154 10.1016/s0165-2478(99)00028-0

[11] Na D, Kim D, Lee D. Mathematical modeling of humoral immune response suppression by passively administered antibodies in mice. J Theor Biol. 2006;241(4):830–51. Epub 2006/03/04. doi: S0022-5193(06)00032-4 [pii] 10.1016/j.jtbi.2006.01.019 . 16513138

[12] Kumpel BM, Elson CJ. Mechanism of anti-D-mediated immune suppression—a paradox awaiting resolution? Trends in Immunology. 2001;22:26–31. 11286688 10.1016/s1471-4906(00)01801-9

[13] Getahun A, Heyman B. Studies on the mechanism by which antigen-specific IgG suppresses primary antibody responses: evidence for epitope masking and decreased localization of antigen in the spleen. Scand J Immunol. 2009;70(3):277–87. doi: SJI2298 [pii] 10.1111/j.1365-3083.2009.02298.x . 19703017

[14] Heyman B, Wiersma E, Nose M. Complement activation is not required for IgG-mediated suppression of the antibody response. Eur J Immunol. 1988;18:1739–43. 3060362 10.1002/eji.1830181113

[15] Sörman A, Zhang L, Ding Z, Heyman B. How antibodies use complement to regulate antibody responses. Mol Immunol. 2014;61(2):79–88. Epub 2014/07/09. doi: S0161-5890(14)00141-2 [pii] 10.1016/j.molimm.2014.06.010 . 25001046

[16] Takai T, Li M, Sylvestre D, Clynes R, Ravetch JV. FcRγ chain deletion results in pleiotrophic effector cell defects. Cell. 1994;76:519–29. 8313472 10.1016/0092-8674(94)90115-5

[17] Molina H, Holers VM, Li B, Fang Y-F, Mariathasan S, Goellner J, et al Markedly impaired humoral immune responses in mice deficient in complement receptors 1 and 2. Proc Natl Acad Sci USA. 1996;93:3357–61. 8622941 10.1073/pnas.93.8.3357 PMC39612

[18] Botto M, Dell'Agnola C, Bygrave AE, Thompson EM, Cook HT, Petry F, et al Homozygous C1q deficiency causes glomerulonephritis associated with multiple apoptotic bodies. Nature genetics. 1998;19(1):56–9. Epub 1998/05/20. 10.1038/ng0598-56 . 9590289

[19] Ey PL, Prowse SJ, Jenkin CR. Isolation of pure IgG1, IgG2a and IgG2b immunoglobulins from mouse serum using protein A-Sepharose. Immunochemistry. 1978;15:429 30693 10.1016/0161-5890(78)90070-6

[20] Jerne NK, Nordin AA. Plaque formation in agar by single antibody-producing cells. Science. 1963;140:405 13957684

[21] Carlsson F, Getahun A, Rutemark C, Heyman B. Impaired antibody responses but normal proliferation of specific CD4+ T cells in mice lacking complement receptors 1 and 2. Scand J Immunol. 2009;70(2):77–84. doi: SJI2274 [pii] 10.1111/j.1365-3083.2009.02274.x . 19630912

[22] Fischer MB, Ma M, Goerg S, Zhou X, Xia J, Finco O, et al Regulation of the B cell response to T-dependent Ags by classical pathway complement. J Immunol. 1996;157:549–56. 8752901

[23] Rutemark C, Alicot E, Bergman A, Ma M, Getahun A, Ellmerich S, et al Requirement for complement in antibody responses is not explained by the classic pathway activator IgM. Proc Natl Acad Sci U S A. 2011;108(43):E934–42. Epub 2011/10/12. doi: 1109831108 [pii] 10.1073/pnas.1109831108 21987785 PMC3203774

[24] Cutler AJ, Botto M, van Essen D, Rivi R, Davies KA, Gray D, et al T cell-dependent immune response in C1q-deficient mice: defective interferon γ production by antigen-specific T cells. J Exp Med. 1998;187:1789–97. 9607920 10.1084/jem.187.11.1789 PMC2212306

[25] Rutemark C, Bergman A, Getahun A, Hallgren J, Henningsson F, Heyman B. Complement receptors 1 and 2 in murine antibody responses to IgM-complexed and uncomplexed sheep erythrocytes. PloS one. 2012;7(7):e41968 Epub 2012/08/01. 10.1371/journal.pone.0041968 PONE-D-12-09317 [pii]. 22848677 PMC3405055

[26] Takai T, Ono M, Hikida M, Ohmori H, Ravetch JV. Augmented humoral and anaphylactic responses in FcγRII-deficient mice. Nature. 1996;379:346–9. 8552190 10.1038/379346a0

[27] Kumpel BM. Lessons learnt from many years of experience using anti-D in humans for prevention of RhD immunization and haemolytic disease of the fetus and newborn. Clin Exp Immunol. 2008;154(1):1–5. Epub 2008/08/30. doi: CEI3735 [pii] 10.1111/j.1365-2249.2008.03735.x 18727626 PMC2561090

[28] Wernersson S, Karlsson M, Dahlström J, Mattsson R, Verbeek JS, Heyman B. IgG-mediated enhancement of Ab responses is low in FcRγ chain deficient mice and increased in FcγRII deficient mice. J Immunol. 1999;163:618–22. 10395649

[29] Bolland S, Ravetch JV. Spontaneous autoimmune disease in FcγRIIB-deficient mice results from strain-specific epistasis. Immunity. 2000;13:277–85. 10981970 10.1016/s1074-7613(00)00027-3

[30] Sinclair NRS. Fc-signalling in the modulation of immune responses by passive antibody. Scand J Immunol. 2001;53:322–30. 11285110 10.1046/j.1365-3083.2001.00889.x

[31] Heyman B, Dahlström J, Diaz de Ståhl T, Getahun A, Wernersson S, Karlsson MCI. No evidence for a role of FcγRIIB in suppression of *in vivo* antibody responses to erythrocytes by passively administered IgG. Scand J Immunol. 2001;53:331–4. 11285111 10.1046/j.1365-3083.2001.00890.x

[32] Bernardo L, Yu H, Amash A, Zimring JC, Lazarus AH. IgG-mediated immune suppression to erythrocytes by polyclonal antibodies can occur in the absence of activating or inhibitory Fc-gamma receptors in a full mouse model. J Immunol. 2015;195(5):2224–30. Epub 2015/07/19. doi: jimmunol.1500790 [pii] 10.4049/jimmunol.1500790 . 26188060

[33] Klaus GGB. Generation of memory cells. III. Antibody class requirements for the generation of B-memory cells by antigen-antibody complexes. Immunology. 1979;37:345–51. 313901 PMC1457494

[34] da Silveira SA, Kikuchi S, Fossati-Jimack L, Moll T, Saito T, Verbeek JS, et al Complement activation selectively potentiates the pathogenicity of the IgG2b and IgG3 isotypes of a high affinitiy anti-erythrocyte autoantibody. J Exp Med. 2002;195:665–72. 11901193 10.1084/jem.20012024 PMC2193744

[35] Nose M, Wigzell H. Biological significance of carbohydrate chains on monoclonal antibodies. Proc Natl Acad Sci USA. 1983;80:6632–6. 6579549 10.1073/pnas.80.21.6632 PMC391224

[36] Duncan AR, Winter G. The binding site for C1q on IgG. Nature. 1988;332:738–40. 3258649 10.1038/332738a0

[37] Karlsson MCI, Diaz de Ståhl T, Heyman B. IgE-mediated suppression of primary antibody responses *in vivo* . Scand J Immunol. 2001;53:381–5. 11285118 10.1046/j.1365-3083.2001.00886.x

