# Supplementary material for: IgG Suppresses Antibody Responses in Mice Lacking C1q, C3, Complement Receptors 1 and 2, or IgG Fc-Receptors
Source: PLoS One. 2015 Nov 30;10(11):e0143841. doi: 10.1371/journal.pone.0143841 (PMC4664261; doi:10.1371/journal.pone.0143841)
Supplement: S1 Fig — C1q KO, C3 KO and C57BL/6 mice were immunized with 50 μg IgGa anti-SRBC and 5x10^7^ SRBC, 5x10^7^ SRBC alone, or with 50 μg IgGa alone. Cr2 KO and BALB/c mice immunized with 50 μg IgGb anti-SRBC and 5x10^7^ SRBC, 5x10^7^ SRBC alone, or with 50 μg IgGb alone. (A-I) Seven-49 days after immunization, serum levels of IgG anti-SRBC were assayed in ELISA on sera diluted 1:625 (A,B,D,E,G,H) or 1:25 (C,F,I). Incubation times with substrate were either 30 min or 3 h. Data are representative of two (A-C, G-I) or one (D-F) experiments; (n = 3-5/group in each experiment). p-values denote comparisons between mice immunized with IgG anti-SRBC together with SRBC and mice immunized with SRBC alone. ns, p > 0.05 (not indicated); *, p < 0.05; **, p < 0.01; ***, p < 0.001. (PDF) [file pone.0143841.s001.pdf]

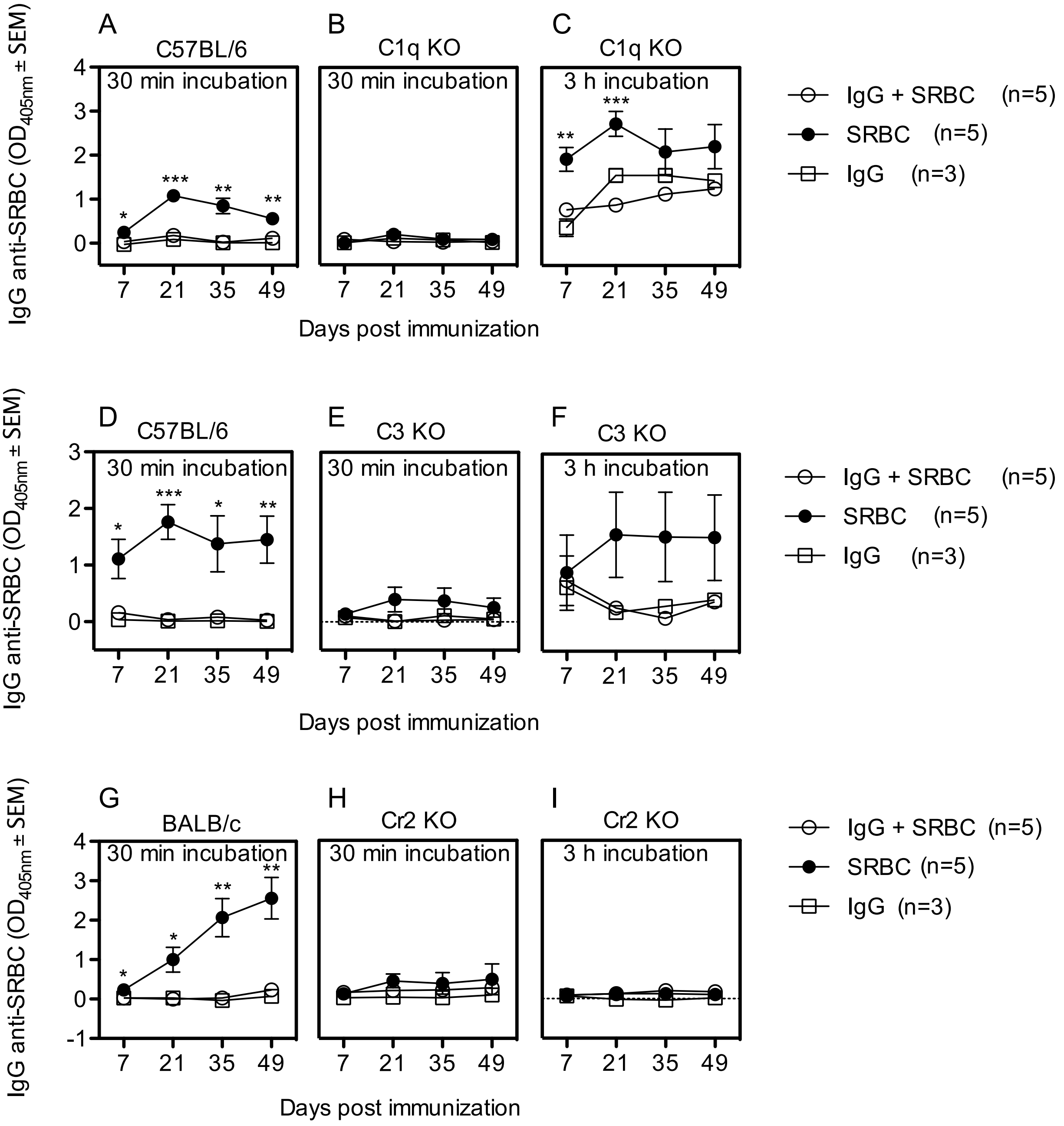

S1 Fig. IgG-mediated suppression of primary IgG-responses in C1q KO, C3 KO and Cr2 KO mice. C1q KO, C3 KO and C57BL/6 mice were immunized with 50 μg IgG[a] anti-SRBC and $5x10^7$ SRBC, $5x10^7$ SRBC alone, or with 50 μg IgG[a] alone. Cr2 KO and BALB/c mice immunized with 50 μg IgG[b] anti-SRBC and $5x10^7$ SRBC, $5x10^7$ SRBC alone, or with 50 μg IgG[b] alone. (A-I) Seven-49 days after immunization, serum levels of IgG anti-SRBC were assayed in ELISA on sera diluted 1:625 (A,B,D,E,G,H) or 1:25 (C,F,I). Data are representative of two (A-C), one (D-F), or two (G-I) experiments; (n=3-5/group in each experiment). p-values denote comparisons between mice immunized with IgG anti-SRBC together with SRBC and mice immunized with SRBC alone. ns, $p > 0.05$ (not indicated); *, $p < 0.05$; **, $p < 0.01$; ***, $p < 0.001$.
